# Study on Maximum Specific Loss Power in Fe_3_O_4_ Nanoparticles Decorated with Biocompatible Gamma-Cyclodextrins for Cancer Therapy with Superparamagnetic Hyperthermia

**DOI:** 10.3390/ijms221810071

**Published:** 2021-09-17

**Authors:** Costica Caizer, Isabela Simona Caizer

**Affiliations:** 1 Department of Physics, Faculty of Physics, West University of Timişoara, 300223 Timişoara, Romania; isabela.caizer@umft.ro; 2 Department of Plastic and Reconstructive Surgery, Faculty of Medicine, “Victor Babes” University of Medicine and Pharmacy of Timişoara, 300041 Timişoara, Romania; 3 Department of Clinical Practical Skills, Faculty of Medicine, “Victor Babes” University of Medicine and Pharmacy of Timişoara, 300041 Timişoara, Romania

**Keywords:** magnetite nanoparticles, γ-cyclodextrins, Néel magnetic relaxation, Brown relaxation, superparamagnetic hyperthermia, maximum specific loss power, alternative cancer therapy

## Abstract

Different chemical agents are used for the biocompatibility and/or functionality of the nanoparticles used in magnetic hyperthermia to reduce or even eliminate cellular toxicity and to limit the interaction between them (van der Waals and magnetic dipolar interactions), with highly beneficial effects on the efficiency of magnetic hyperthermia in cancer therapy. In this paper we propose an innovative strategy for the biocompatibility of these nanoparticles using gamma-cyclodextrins (γ-CDs) to decorate the surface of magnetite (Fe_3_O_4_) nanoparticles. The influence of the biocompatible organic layer of cyclodextrins, from the surface of Fe_3_O_4_ ferrimagnetic nanoparticles, on the maximum specific loss power in superparamagnetic hyperthermia, is presented and analyzed in detail in this paper. Furthermore, our study shows the optimum conditions in which the magnetic nanoparticles covered with gamma-cyclodextrin (Fe_3_O_4_–γ-CDs) can be utilized in superparamagnetic hyperthermia for an alternative cancer therapy with higher efficiency in destroying tumoral cells and eliminating cellular toxicity.

## 1. Introduction

In alternative cancer therapy, magnetic hyperthermia and/or superparamagnetic hyperthermia has been used with very promising results in vitro and in vivo [1,2,3,4,5,6,7,8,9,10,11,12,13,14,15,16,17,18]. These therapies consist of the heating of magnetic nanoparticles to 42–43 °C under the action of an alternating magnetic field with a frequency of 100–1000 kHz. The first type of magnetic hyperthermia uses the hysteresis effect obtained in the magnetic field to heat nanoparticles [2], an effect that is achieved in large nanoparticles, generally more significant than 20–25 nm [19]. The second type of magnetic hyperthermia uses the magnetic relaxation effect, which is achieved for small nanoparticles in the magnetic field, generally <20 nm. However, the size of magnetic nanoparticles and the obtained effect after the magnetic field is applied will also depend significantly on the magnetic anisotropy, which leads to a classification from this point of view in two distinct classes for the magnetic nanomaterials: (i) soft, in the case with small anisotropy (generally in the (1–50) × 10^3^ Jm^−3^ range), and (ii) hard, in the case of those with higher or very considerable anisotropy (10^5^–10^6^ Jm^−3^).

In this paper, we consider the soft magnetite (Fe_3_O_4_) nanoparticles with a magnetocrystalline anisotropy constant of 11 × 10^3^ Jm^−3^, which is the most commonly used in magnetic hyperthermia and with the best results in alternative cancer treatment [7,9,12,13,14,15,17,18,20,21]. For small nanoparticles of Fe_3_O_4_, under 20 nm, their magnetic structure is single domain [19,22,23], and in an external magnetic field and at room temperature the nanoparticles behave superparamagnetic [24,25]. As a result, the magnetic hyperthermia effect in this case is obtained through the superparamagnetic relaxation effect [25,26]; this type of hyperthermia is known as superparamagnetic hyperthermia. The heating of the nanoparticles in this case is produced through the Néel magnetic relaxation [19,25] due to the rotation of magnetic moments from the inside of nanoparticles when these are fixed. In the case of the dispersion of nanoparticles in a liquid, such as ferrofluid [27], alongside the rotation of the magnetic moment of nanoparticles, the rotation of the nanoparticle itself as a result of thermal agitation/activation also takes place, giving the Brown relaxation effect [26,28]. At a given temperature (for example, room temperature) the Brown relaxation depends on the viscosity of the environment in which the nanoparticles are dispersed, and also on the size (volume) of the nanoparticles, the effect being directly proportional with these variables. In the case of spherical nanoparticles, where the volume (V) expressed through their diameter (D) is dependent on the 3rd power (V = πD^3^/6), there will exist a strong dependence between Brown relaxation and the diameter of the nanoparticles.

Compatibilizing the surface of magnetic nanoparticles by decorating them with organic molecules, with the aim of eliminating any of their toxicity upon health tissues, will increase their diameter depending on the molecular chain length used for different organic structures. In this case it will be considered a hydrodynamic diameter (D_h_) [29] given by the formula D_h_ = D + 2d in the spherical nanoparticles case, where d is the thickness from the surface of the nanoparticles. In practice, the thickness of the organic layer (d) from the surface of the nanoparticles can vary in a large range, from ~1 nm to tens or even hundreds of nm depending on the organic agents used for biocompatibilization or biofunctionalization of magnetic nanoparticles. These will radically change the relaxation effect, with direct consequences on the loss power in superparamagnetic hyperthermia and, in the end, on the heating temperature of nanoparticles. Thus, these relaxation effects (Néel and Brown) will reflect on superparamagnetic hyperthermia efficiency and effectiveness for thermally destroying tumor cells.

With a view to the elimination of the cellular toxicity of Fe_3_O_4_ nanoparticles and also the elimination of the van der Waals and magnetic dipolar interactions that lead to nanoparticle agglomeration in large structures, with negative effects on superparamagnetic hyperthermia; in this paper we proposed biocompatibilization of the Fe_3_O_4_ nanoparticles through the decoration of their surface with gamma-cyclodextrins (γ-CDs) [30,31], for a significant beneficial effect on magnetic hyperthermia. Cyclodextrins (CDs) represent a highly interesting class of biocompatible systems. CDs are nontoxic cyclic oligosaccharide nano-objects consisting of α(1-4)-linked–d-glucose units. The natural γ-CDs consist of eight glucopyranose units [30]. These compounds maintain a high solubility in aqueous media due to their hydrophilic terminations. Nanoparticles can be functionalized with CDs with a wide potential in biomedical applications [32], which could provide a target-delivery of NPs. The advantages of bioconjugating magnetic nanoparticles with CDs are: (a) lack of toxicity, (b) stability of final bionanostructures, (c) possibility of an easy administration (by direct injection), and (d) much higher quantity of nanoparticles (or active substance) towards the tumor site (the thickness of the CDs layer covering the nanoparticles being only 0.8 nm, the concentration of bionanoparticles dispersed in the suspension to be administered can be much increased). Thus, as cyclodextrins are perfectly biocompatible with oligosaccharides, the nanoparticles covered with cyclodextrins (Fe_3_O_4_–γ-CDs) will be non-toxic [32].

Taking all these aspects into account, in this paper we present a study about the influence of the organic layer of gamma-cyclodextrins on the maximum specific loss power (P_sM_) in superparamagnetic hyperthermia. Furthermore, finding the optimum conditions in which superparamagnetic hyperthermia with biocompatible magnetite nanoparticles of Fe_3_O_4_–γ-CDs can be used in order to obtain the maximum efficiency in destroying tumor cells, is also presented. Finally, the influence of γ-CDs layers on Néel magnetic relaxation, Brown relaxation, and loss power in superparamagnetic hyperthermia is analyzed and discussed in detail.

## 2. Basic Equations Used in the Calculation of Specific Loss Power in the Case of Biocompatible Magnetic Nanoparticles Dispersed in a Liquid

In magnetic hyperthermia, in order to introduce the biocompatible magnetic nanoparticles in the tumor using different techniques (direct injection into the localized tumor, injection into the blood vessel which feeds the tumor, etc.), they need to be dispersed in a base-liquid (pharmaceutical, physiological serum, deionized water, etc.), according to a certain nanoparticle concentration (or magnetic packing fraction (ε)), that can reach up to 10–30 mg/mL [33].

Due to thermal agitation at temperature T (e.g., room temperature) the nanoparticles can rotate in the liquid giving the known Brown relaxation effect [26,28]. This relaxation process is quantitatively characterized at temperature T by the Brown relaxation time:

(1) $$\tau_{B\;}=\frac{3\pi \eta D^{3}}{6k_{B}T}\;\;\;\;$$

where *D* is the physical diameter of spherical nanoparticles, *η* is the viscosity coefficient of the dispersion liquid, and *k_B_* is Boltzmann’s constant (1.38 × 10^−23^ J/molK). For a used liquid, it is important to remember that this relaxation time increases strongly (the relaxation effect intensifies) with the increase of the diameter (size) of nanoparticle, and with the decrease of temperature.

When the nanoparticles are biocompatibilized by decorating their surfaces with different organic structures a hydrodynamic diameter [29,34] instead of physical diameter (*D*) of nanoparticles must be taken into consideration, given by the formula

(2) $$D_{h}=D+2d\;\;\;\;\;$$

where *d* is the thickness of organic layer from the surface of the nanoparticles. Thus, the Brown relaxation time in this case will be:

(3) $$\tau_{B\;}=\frac{3\pi \eta {\left(D+2d\right)}^{3}}{6k_{B}T}\;\;\;$$

Moreover, also taking place at *T* temperature is the rotation of the magnetic moment of superparamagnetic nanoparticles [24]. This process is quantitatively characterized by the Néel relaxation time [25]:

(4) $$\tau_{N}=\tau_{0}\cdot exp\left(\frac{\pi KD^{3}}{6k_{B}T}\right)$$

where, besides the nanoparticle size (*D*), this depends exponentially on the magnetic anisotropy constant *K* of the nanoparticles. The observable *τ*_0_ is a time constant that is usually 10^−9^ s [35].

As a function of the relation in which the Néel and Brown relaxation times can be found, there may be situations in which only the Néel relaxation time or Brown relaxation time can exist, or both when they are similar values. When both Néel and Brown relaxation processes exist in the nanoparticles system, the total relaxation time will be considered, given by the formula [36]

(5) $$\frac{1}{\tau }=\frac{1}{\tau_{N}}+\frac{1}{\tau_{B\;}}\;$$

and

(6) $$\tau =\frac{\tau_{N\;}\tau_{B\;}}{\tau_{N\;}+\tau_{B\;}}\;\;\;\;$$

respectively.

In these conditions, in the case of dispersed biocompatible magnetic nanoparticles of Fe_3_O_4_–γ-CDs used in superparamagnetic hyperthermia, when the Brown relaxation time is present alongside the Néel relaxation time, the specific loss power obtained after applying the external alternating magnetic field with *H* amplitude and *f* frequency [37] will be:

(7) $$P_{s}=\frac{3\pi \mu_{0}\chi_{i}}{\rho \xi }\left(\coth\xi -\frac{1}{\xi }\right)\frac{2\pi f\frac{\tau_{N\;}\tau_{B\;}}{\tau_{N\;}+\tau_{B\;}}}{1+{\left(2\pi f\frac{\tau_{N\;}\tau_{B\;}}{\tau_{N\;}+\tau_{B\;}}\right)}^{2}}fH^{2}\;\;\;\;\;\;\left(W/g\right)$$

where

(8) $$\chi_{i}=\frac{\varepsilon \pi \mu_{0}M_{s}^{2}D^{3}}{18k_{B}T}$$

and

(9) $$\xi =\frac{\pi \mu_{0}M_{s}D^{3}}{6k_{B}T}H\;\;$$

In this case the relaxation time *τ*_B_ is given by Equation (3) and not by Equation (1). In Equation (7), the dependence of the static magnetic susceptibility of nanoparticles on magnetic field amplitude is considered [37]. In Equations (7)–(9), the observables are the following: *χ*_i_ is the initial magnetic susceptibility of the magnetic nanoparticles, *ξ* is the Langevin parameter [24,38,39] given by Equation (9), ρ is the density material, M_s_ is the spontaneous magnetization, *ε* is the volume packing fraction, and *μ*_0_ is the magnetic permeability of the vacuum. The magnetic packing fraction considers the concentration of the magnetic nanoparticles in the whole liquid volume.

In conclusion, in the case of Fe_3_O_4_–γ-CDs bionanoparticles dispersed in a liquid, the specific loss power will be calculated with Equation (7), where the observables given by Equations (8) and (9) and those given by Equations (3), (4) and (6), are used.

## 3. Results and Discussion

### 3.1. Biocompatible Fe_3_O_4_ Nanoparticles Decorated with γ-CDs and Dispersed in a Liquid to Eliminate Toxicity and Increase Efficiency in Superparamagnetic Hyperthermia

For the safe use magnetic nanoparticles in malignant tumor therapy through superparamagnetic hyperthermia, the nanoparticles must be biocompatible with the biological tissue in which they will be introduced, using different techniques from modern nanobiotechnology: surfactation, encapsulation, decoration, biofunctionalization, etc. Small nanoparticles of Fe_3_O_4_ (just a few nm) and low concentration of nanoparticles in suspensions (~μg/mL to hundreds μg/mL) are non-toxic for the biological tissue [40]. However, to improve the magnetic hyperthermia efficiency, there is a need to increase the concentration of nanoparticles in suspensions up to 10–30 mg/mL [33] or higher. Further, the loss power in superparamagnetic hyperthermia increases at larger values of the nanoparticles, respectively ~16 nm as we showed in Ref. [37]. Thus, in these conditions there may be some cellular toxicity, depending on multiple factors, including the nature of the biological tissue. Therefore, in order to reduce the cellular toxicity or even eliminate it, nanoparticles must be made biocompatible using different chemical agents [40,41,42].

Thus, taking into consideration the beneficial advantages of using cyclodextrins in pharmacology, food, etc., and also on the hyperthermia effect [31], we propose using gamma-cyclodextrins (γ-CDs) for biocompatibilization of magnetite (Fe_3_O_4_) nanoparticles, which give the maximum effect in superparamagnetic hyperthermia. The preparation of nanoparticles in the presence of a cyclodextrin leads to *core-shell* bionanostructures with the oligosaccharide, with the same solubility properties as the γ-CDs itself. CDs are currently proposed as drugs carriers in pharmacology due to their lack of toxicity and possibility to load hydrophobic molecules (i.e., drugs or extracts) in their toroidal cavity. Due to this, we propose their use as possible nanocarriers for magnetic nanoparticles towards targeted cancer cells without involving toxicity.

**Figure 1:**
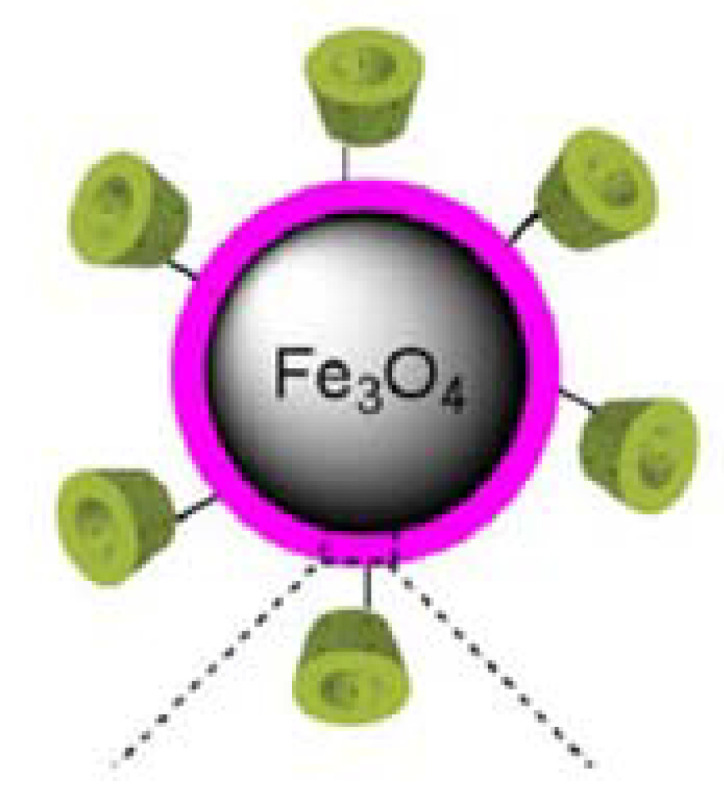
β-CD-functionalized magnetic nanoparticles (β-CD-PDA-MNPs) [43]. © 2017 Elsevier B.V.

**Figure 2:**
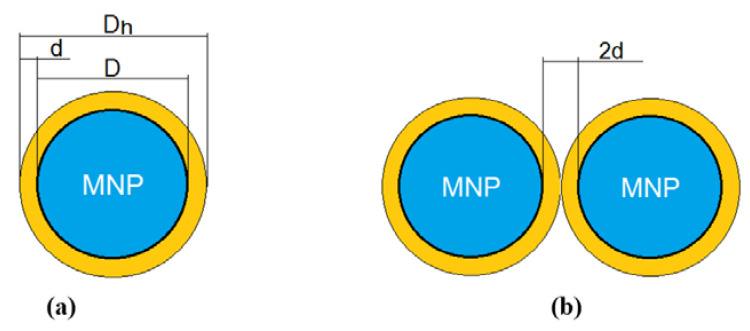
(**a**) The core-shell model of the magnetic Fe_3_O_4_-γ-CDs bionanoparticle: the core is the magnetic Fe_3_O_4_ nanoparticle with the diameter D, and the shell is the organic layer from the surface (γ-CDs with biopolymer) with the thickness d; D_h_ is the hydrodynamic diameter of the bionanoparticle; (**b**) bionanoparticles system with the minimum distance between them 2d for evaluating the maximum interactions.

By covering the surface of nanoparticles with γ-CDs, as in Figure 1 for β-CD [43], using an appropriate binder agent, such as the polyacrylic acid (PAA) [44], a hybrid core-shell magnetic nanobiostructure of Fe_3_O_4_–γ-CDs is obtained, where the core is Fe_3_O_4_, and the shell gives the thickness of the organic layer (CDs and binding agent). This bionanostructure can be schematically represented as in Figure 2a, where the magnetite nanoparticle has a D diameter and the thickness of the organic layer from the surface is given by the thickness d.

Taking into consideration the molecular size of γ-CDs of 8 °A and that of the binding polymer thickness of ~0.8 nm (determined through DLS [44]), we will consider in our study the thickness d = 1.6 nm (Table 1). Thus, the hydrodynamic diameter D_h_ will be given by Equation (2).

From the point of view of superparamagnetic hyperthermia, using γ-CDs to biocompatibilization of Fe_3_O_4_ nanoparticles presents two significant benefits: (i) the lack of cellular toxicity, cyclodextrins being without toxicity for the organism [31]; and (ii) eliminating van der Waals and dipole–dipole interactions between nanoparticles [27,45,46]. This will allow for the maintenance of the nanoparticles in suspension as individual nanoparticles, separated one from the other by at least 2d (Figure 2b), and forming stable suspensions in time. Furthermore, the van der Waals and the magnetic dipole interaction are negligible because the magnetic nanoparticles have at least 2d, or 3.2 nm, distance between them [46,47]. Moreover, (iii) in the case of using γ-CDs, the thickness of the surface layer of the nanoparticles will remain only 1.6 nm [44], allowing an accentuated increase of the concentration of the magnetic nanoparticles in suspension (increasing the magnetic packing fraction). Thus, it is possible to exceed 10–30 mg/mL concentration, leading to the possibility of increasing the specific loss power compared with other larger biostructures, where the organic layer can have tens or even hundreds of nm, as in the case of liposomes [9,18,48]. In this last example, the volume magnetic fraction will decrease significantly, leading to a decrease of the maximum specific loss power as well, including the hyperthermia effect on tumors.

In conclusion, by using γ-CDs the efficiency and efficacy of superparamagnetic hyperthermia will increase significantly, concomitant with the elimination of both cellular toxicity and the interactions between the nanoparticles, with huge benefits for magnetic hyperthermia.

In order to administer the bionanoparticles of Fe_3_O_4_–γ-CDs in the tumor they will be dispersed in a pharmaceutical liquid (saline).

The characteristic observables of the magnetic nanoparticles of Fe_3_O_4_ decorated with γ-CDs that will be used in our study are presented in Table 1. The parameters of the alternating magnetic field that will be used are also presented.

### 3.2. Maximum Specific Loss Power in Fe_3_O_4_ Nanoparticles Decorated with γ-CDs Dispersed in a Liquid

For the study of maximum specific loss power in the case of superparamagnetic hyperthermia with Fe_3_O_4_–γ-CDs bionanoparticles, we computed it with Equation (7) with the variables in Equations (3), (4), (6), (8) and (9) in the case of nanoparticles dispersed in a pharmaceutical liquid (saline), considering a volume magnetic packing fraction (ε) of 0.024 (usual value). However, taking into consideration the observations shown in Section 3.1, in our bionanoparticles case the magnetic packing fraction can still increase significantly. We undertook our study taking into consideration the size of nanoparticles (D diameter), a critical variable in magnetic hyperthermia [51], and the parameters of the alternating magnetic field, frequency (f), and amplitude (H), in a specific magnetic hyperthermia values range. To analyze the data obtained, we used a 3D representation (simultaneous of two variables) on extended domain values (Table 1) in order to capture the changes of specific loss power and maximum values as a function of different parameters in the study.

**Figure 3:**
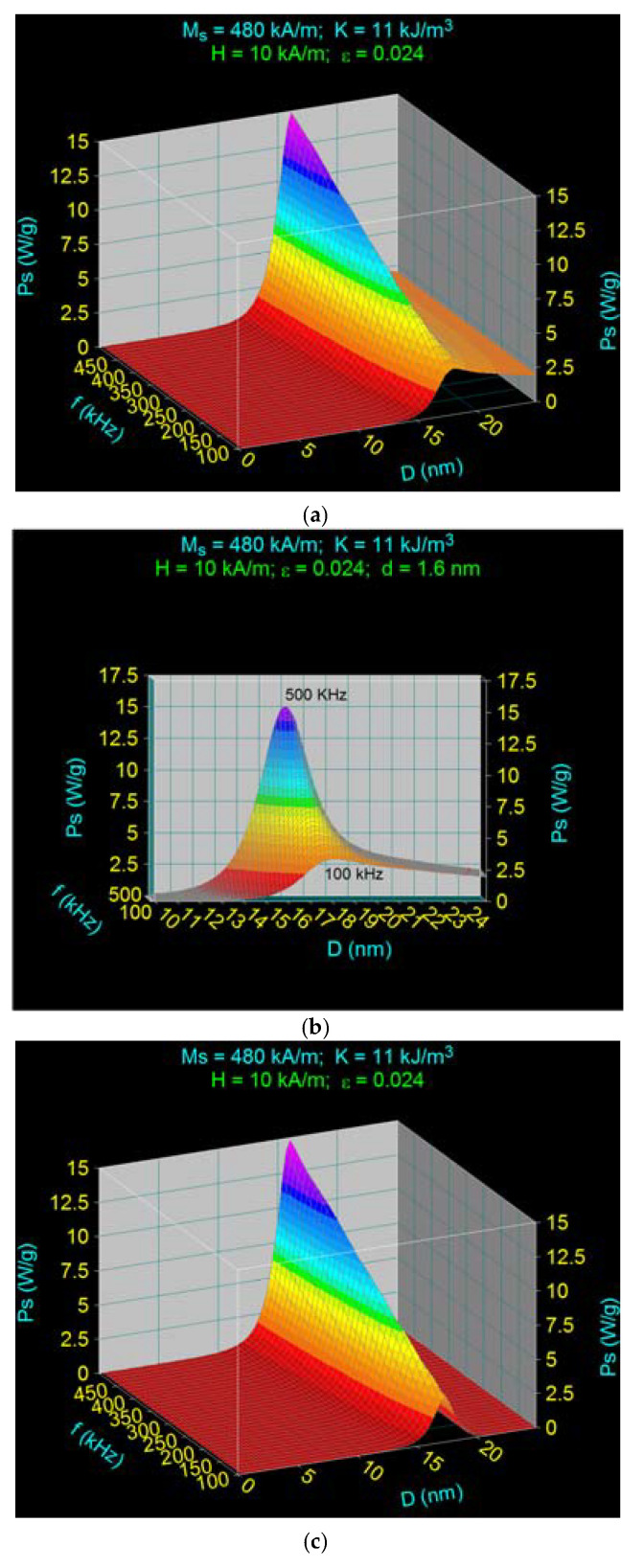
Specific loss power as a function of nanoparticle diameter and magnetic field frequency for Fe_3_O_4_–γ-CDs bionanoparticles dispersed in saline in (**a**) 3D and (**b**) 2D cases, and (**c**) for Fe_3_O_4_ nanoparticles.

Thus, using the characteristic observables in Table 1, the specific loss power in the case of Fe_3_O_4_–γ-CDs bionanoparticles dispersed in liquid calculated as a function of the diameter D of nanoparticles and the frequency f of magnetic field, is shown in 3D in Figure 3a for the magnetic field amplitude of 10 kA/m. In Figure 3b is shown the front view of the specific loss power variation for the diameter range limited at 10–25 nm, which is of interest, and Figure 3c shows the specific loss power calculated in the same conditions as in Figure 3a but lacking the cyclodextrins on the surface of fixed nanoparticles.

Results obtained show the following:

In Figure 3a, the presence of a narrow maximum of specific loss power at the frequency of 500 kHz can be observed, and two overlay maximums comparable as values at a low frequency of 100 kHz (Figure 3b).

In contrast, in Figure 3c, compared with Figure 3a,b, there is only a single maximum of specific loss power at any frequency. This maximum at the 500 kHz frequency corresponds to the maximum in Figure 3a obtained for ~16 nm diameter. Results are in agreement with those obtained in [37] for the specific loss power in the case of Fe_3_O_4_ nanoparticles in the absence of any organic layer at their surface. This result confirms the fact that the narrow maximum from Figure 3a is determined by Néel magnetic relaxation. The maximum from Figure 3a, as with that from Figure 3c, decreases concurrently with the decreasing magnetic field frequency until 100 kHz, and also moves slowly to a more significant diameter value, arriving at the value of 18.1 nm for the frequency of 100 kHz, as we have previously found [37].

Taking into account this result, we can say that the second maximum from Figure 3a,b at 100 kHz is determined by the Brown relaxation, as a result of the presence of the organic layer of γ-CDs with 1.6 nm thickness at the surface of the nanoparticles and the rotation move of biocompatible Fe_3_O_4_–γ-CDs in liquid. This Brown maxim is slightly masked by the presence of the Néel maxim. Furthermore, Brown relaxation being independent of the magnetic field frequency, the maximum specific loss power remains unchanged with increasing frequency from 100 kHz to 500 kHz (Figure 3a,b).

**Figure 4:**
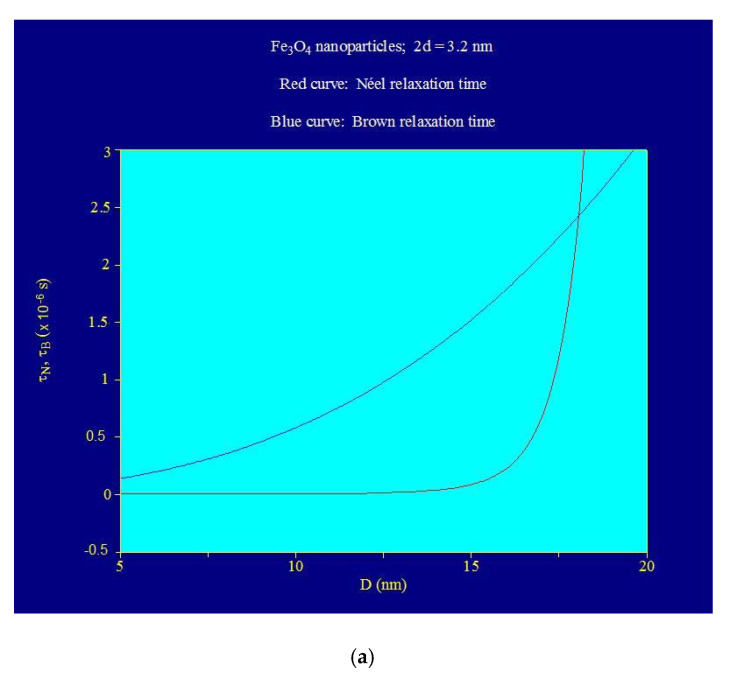

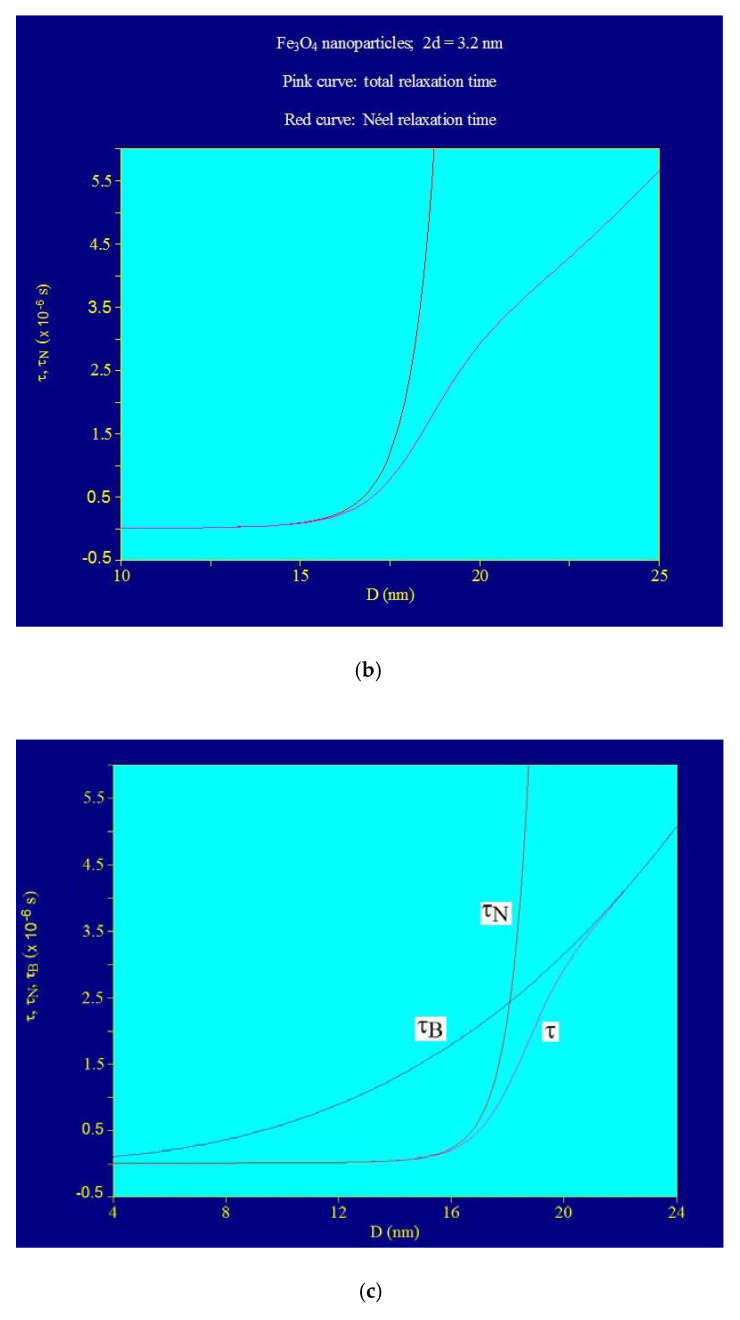
Relaxation times as a function of diameter of Fe_3_O_4_ nanoparticles covered with γ-cyclodextrins: (**a**) Néel and Brown relaxation times, (**b**) Néel and total relaxation times, (**c**) Néel relaxation time, Brown relaxation time, and total relaxation time.

These are confirmed by the variations of Néel magnetic relaxation time and Brown relaxation time depending on the Fe_3_O_4_–γ-CDs bionanoparticles diameter, presented in Figure 4. The variation of Néel magnetic relaxation time (Equation (4)) is determined by the D physical diameter of Fe_3_O_4_ nanoparticle, and Brown relaxation time variation (Equation (3)) is determined by D_h_ hydrodynamic diameter (Equation (2)) of the Fe_3_O_4_–γ-CDs bionanoparticles (Figure 2a).

From Figure 4, results are clear that until ~16 nm diameter Néel relaxation time prevails, where Néel relaxation time is much smaller than Brown relaxation time (Figure 4a). At ~17 nm diameter value, the Néel and Brown relaxation times become comparable, and at ~17.5 nm these become even equal, thus in this range of diameters the relaxation takes place through both Néel and Brown processes. For diameters larger than ~18 nm the Brown relaxation prevails, Brown relaxation time being shorter than the Néel relaxation time that nevertheless increases significantly (exponentially) in this area (Figure 4a,c). The variation of the total relaxation time τ from Figure 4b shows these aspects. From Figure 4c, it can be observed that for nanoparticles under 16 nm diameter, the total relaxation time is determined just by the Néel component, and at >20 nm diameters, the total relaxation time is determined only by the Brown component. In the interval ~16–20 nm (Figure 4c), both relaxation processes are contributing.

From a superparamagnetic hyperthermia point of view, the result in Figure 3a is significant, showing that the contributions of Néel and Brown relaxation processes at the maximum specific loss power depend on the frequency. For example, if magnetic hyperthermia should be achieved at a 500 kHz frequency, then the diameters of the nanoparticles should be 16.1 nm to achieve maximum power, and hyperthermia is determined in this case by the Néel relaxation processes (Figure 3a,b).

Nevertheless, if magnetic hyperthermia is achieved at 100 kHz (Figure 3b), maximum loss power is obtained in an extensive diameter interval, starting from ~17 nm until 25–30 nm or even more, the power decreases slowly along with the increasing diameter. This result can be a real advantage from a practical point of view: it indicates the use of nanoparticles bigger than 16 nm to obtain magnetic hyperthermia, and indicates that the specific loss power decreases only slightly with increasing diameters of the nanoparticles. This result becomes essential from a practical point of view in the case of the distribution of nanoparticles sizes where a strict diameter of 16.1 nm cannot be achieved through the preparation methods. In this last case, the specific loss power and the heating of nanoparticles in superparamagnetic hyperthermia are done mainly by Brown relaxation processes.

### 3.3. The Effect of the Amplitude and Frequency of the Magnetic Field on the Maximum Specific Loss Power in Fe_3_O_4_ Nanoparticles Decorated with Gamma-Cyclodextrins

#### 3.3.1. The Effect of the Amplitude of the Magnetic Field

**Figure 5:**
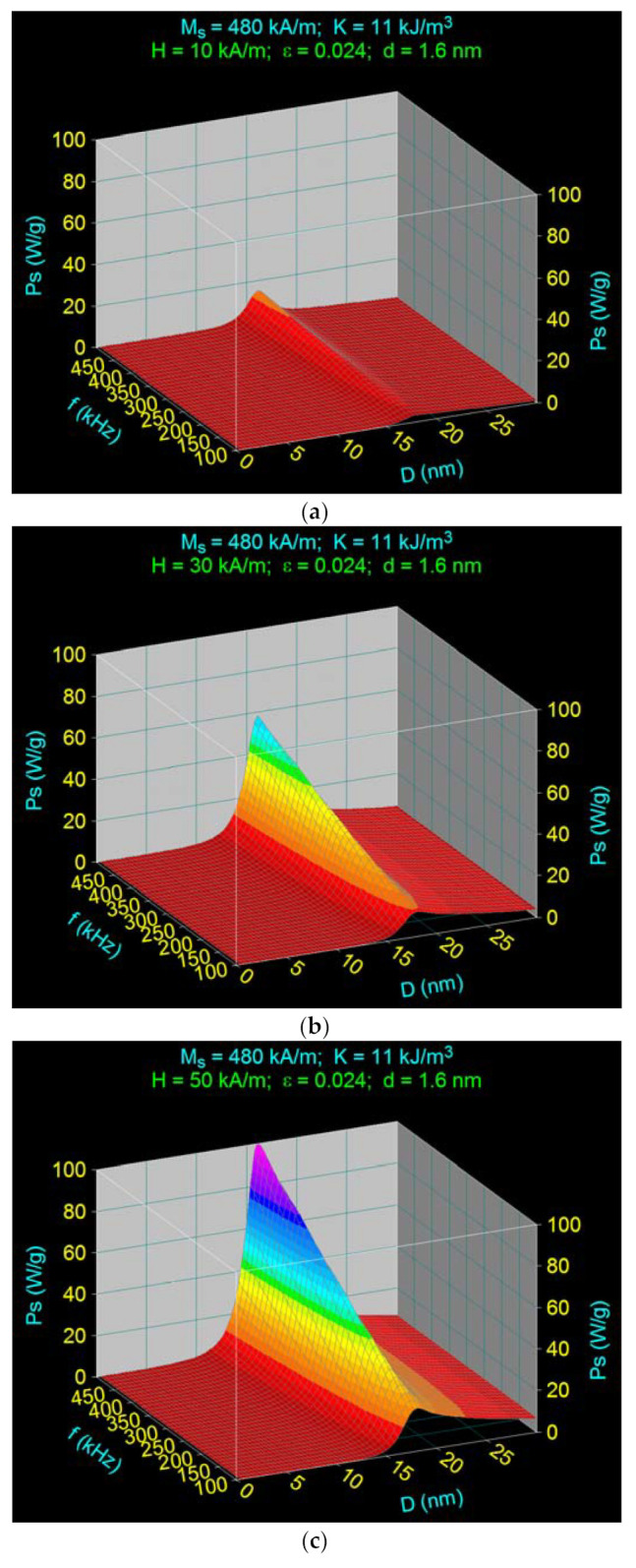
Specific loss power in the case of dispersed Fe_3_O_4_–γ-CDs bionanoparticles for different amplitudes of magnetic field: (**a**) 10 kA/m, (**b**) 30 kA/m, (**c**) 50 kA/m.

Another important aspect of using Fe_3_O_4_–γ-CDs bionanoparticles in superparamagnetic hyperthermia is how the amplitude of the magnetic field influences magnetic hyperthermia. For this study we calculated and 3D registered the specific loss power for dispersed Fe_3_O_4_–γ-CDs bionanoparticles considering a magnetic field variation in the 10–50 kA/m range. In Figure 5 are presented the specific loss powers depending on the nanoparticles diameter and frequency in the 100–500 kHz range for three values of the amplitude of magnetic field. Furthermore, Table 2 presents the maximum specific loss power and diameters values achieved for the nanoparticles’ diameters corresponding with the maximums for frequencies of 100 kHz and 500 kHz.

The obtained results show an important aspect: the position of the maximum of specific loss power in relation to the diameter of the nanoparticles (D_M_) in the case of dispersed Fe_3_O_4_–γ-CDs bionanoparticles does not change for different amplitudes of the magnetic field (10–50 kA/m).

The maximum specific loss power is obtained at the same diameter values of the nanoparticles for a frequency ~16.1 nm at 500 kHz, and ~18 nm at 100 kHz (Table 2). Furthermore, maximum specific loss power increases significantly with an increase in the amplitude of magnetic field (Figure 5, Table 2).

**Figure 6:**
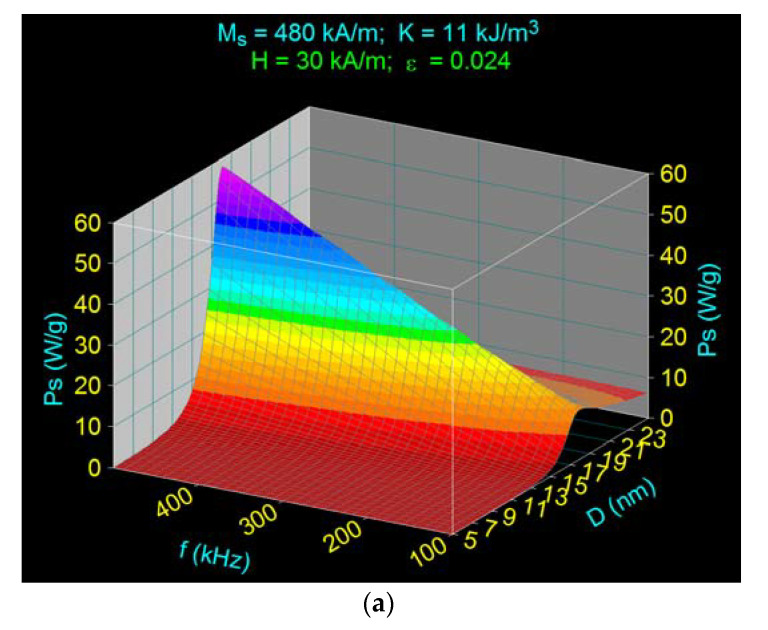

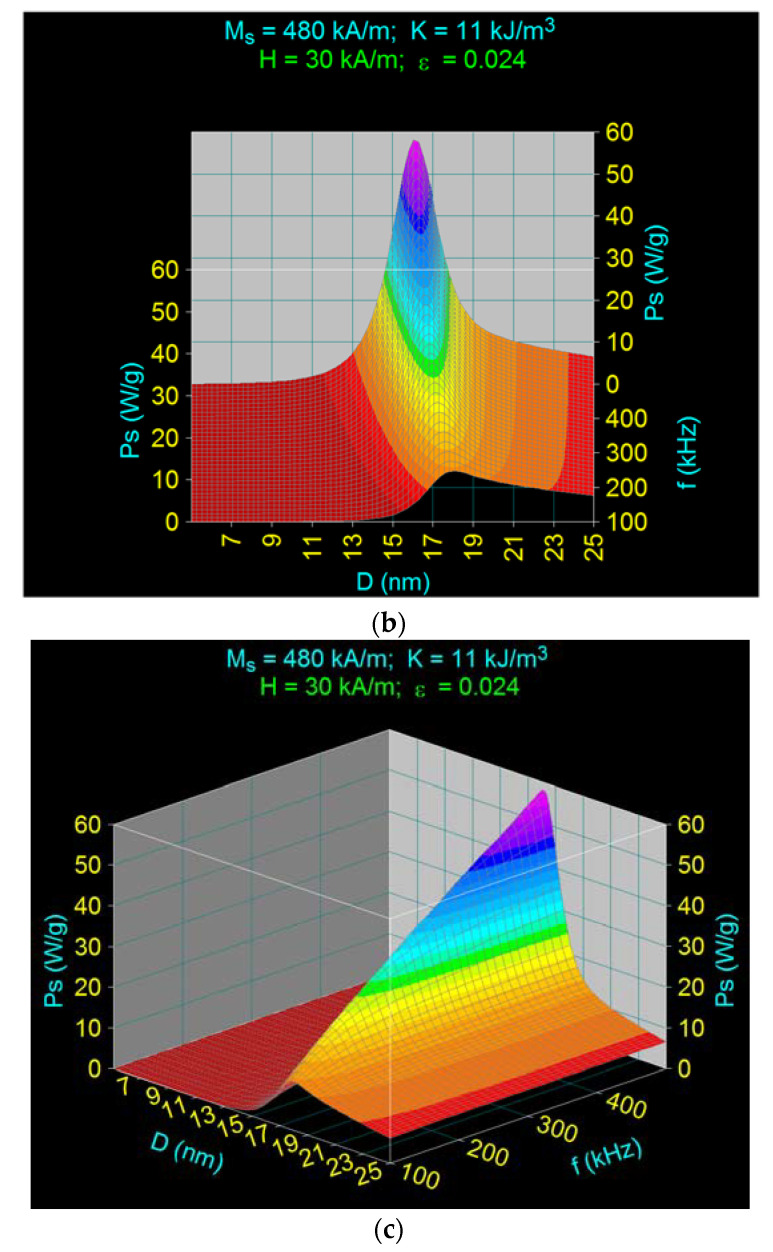
The contribution of the Néel and Brown relaxation processes to the maximum specific loss power in the case of dispersed bionanoparticles of Fe_3_O_4_–γ-CDs for the diagrams at (**a**) D < D_M_, (**b**) front, (**c**) D > D_M_.

**Figure 7:**
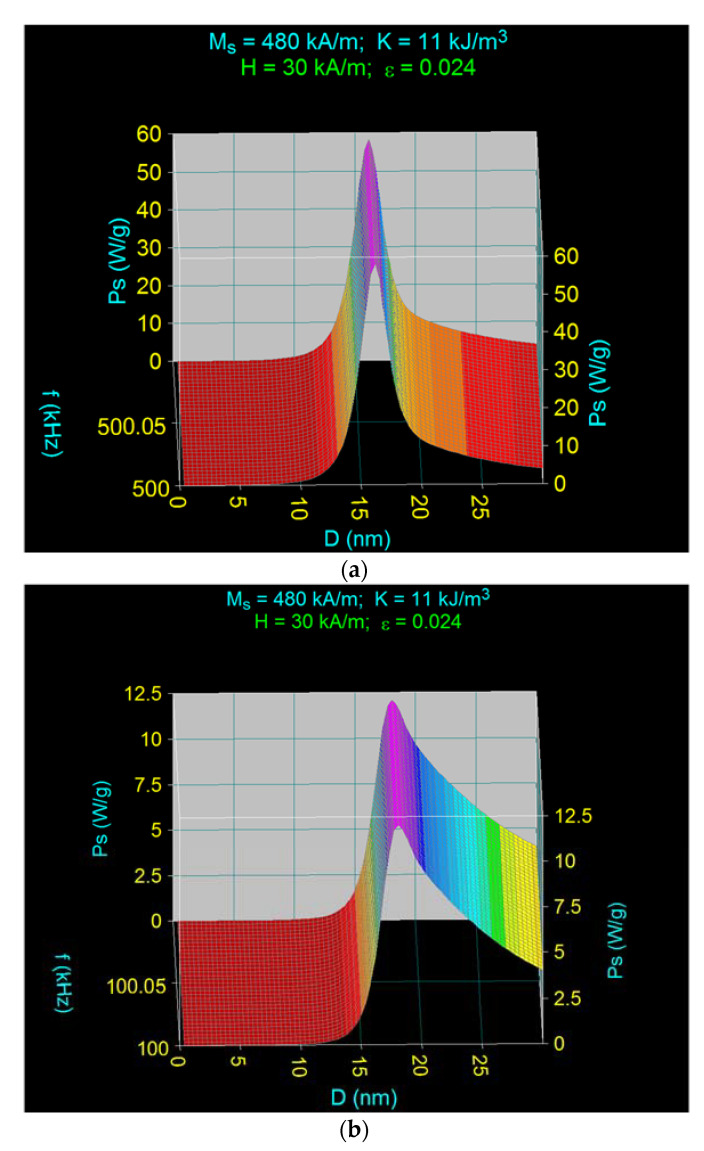
The contribution of the Néel and Brown relaxation processes to the maximum specific loss power in the case of dispersed Fe_3_O_4_–γ-CDs bionanoparticles for (**a**) 500 kHz, and (**b**) 100 kHz.

At 500 kHz frequency, the maximum specific loss power reaches the value of the nanoparticle diameter both in Fe_3_O_4_–γ-CDs nanoparticles and in Fe_3_O_4_ uncovered nanoparticles. This is explained by the fact that the maximum specific loss power in Fe_3_O_4_–γ-CDs nanoparticles is determined by just the Néel relaxation, the Brown relaxation being practically negligible (Figure 6 and Figure 7a).

Decreasing the frequency from 500 kHz to 100 kHz (Figure 7) shows that the maximum specific loss power shifts at ~18.0 nm in diameter (Figure 7b and Table 2). This result obtained at a 100 kHz frequency differs from the one obtained in [37] for Fe_3_O_4_ nanoparticles at the same frequency, where the nanoparticle diameter for the maximum specific loss power is 17.3 nm, and not 18.0 nm as in this case. Increase of the diameter in the present case is due to the significant contribution of the Brown relaxation process that overlaps with the Néel relaxation process at the 100 kHz frequency (Figure 7b), this leads to a shift of the maximum specific loss power at higher diameter values of the nanoparticles. The maximum due to Brown relaxation comparable with that given by Néel relaxation is very wide, extending to higher values of the diameter up to 25–30 nm, where it still has a significant value (Figure 7b). This determines the maximum specific loss power to be obtained at a diameter of 18.0 nm and not at 17.3 nm.

**Figure 8:**
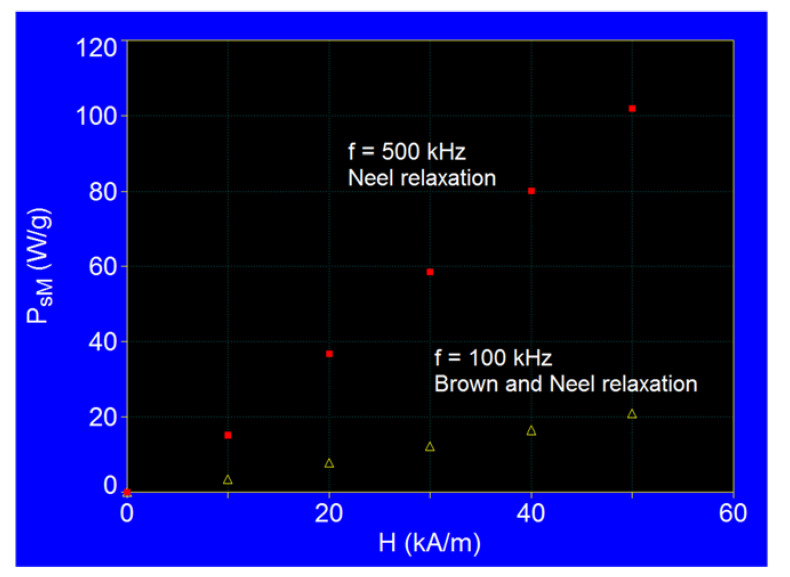
The maximum specific loss power in dispersed Fe_3_O_4_–γ-CDs bionanoparticles as a function of the amplitude of magnetic field.

The maximum specific loss powers determined at both the frequency of 500 kHz and 100 kHz (Figure 5) for the values of the magnetic field in the range of 10–50 kA/m, are shown in Figure 8. The results show that the maximum specific loss power increases almost linearly with the amplitude of the magnetic field, the slope being significantly higher at the frequency of 500 kHz. At the same time, the maximum specific loss power at 500 kHz is narrow, being determined by the Néel relaxation processes, and increases quite rapidly with the amplitude of the magnetic field. However, the maximum specific loss power at 100 kHz is wide, being determined by both Néel and Brown relaxation processes, and increases slowly with the amplitude of the magnetic field.

For other values of the frequency in the range 100–500 kHz, the values of the maximum specific loss power are between the values determined by the square and triangle points (Figure 8), with the remark that as the frequency increases from 100 kHz to 500 kHz the contribution of Néel relaxation at the maximum power increases compared with Brown relaxation, until the Brown relaxation process becomes negligible at frequencies of 400–500 kHz.

#### 3.3.2. The Effect of the Magnetic Field Frequency

The maximum values of the specific loss power and the values of the diameters of nanoparticles determined when the frequency increases in the range of 100–500 kHz (Figure 6a,b) are given in Table 3. These were determined for the magnetic field of 10 kA/m. For other values of the magnetic field the values of the diameters corresponding to the maximums of the specific loss power do not change; only the values of the maximum specific loss power change, increasing with the amplitude of magnetic field (Figure 8).

**Figure 9:**
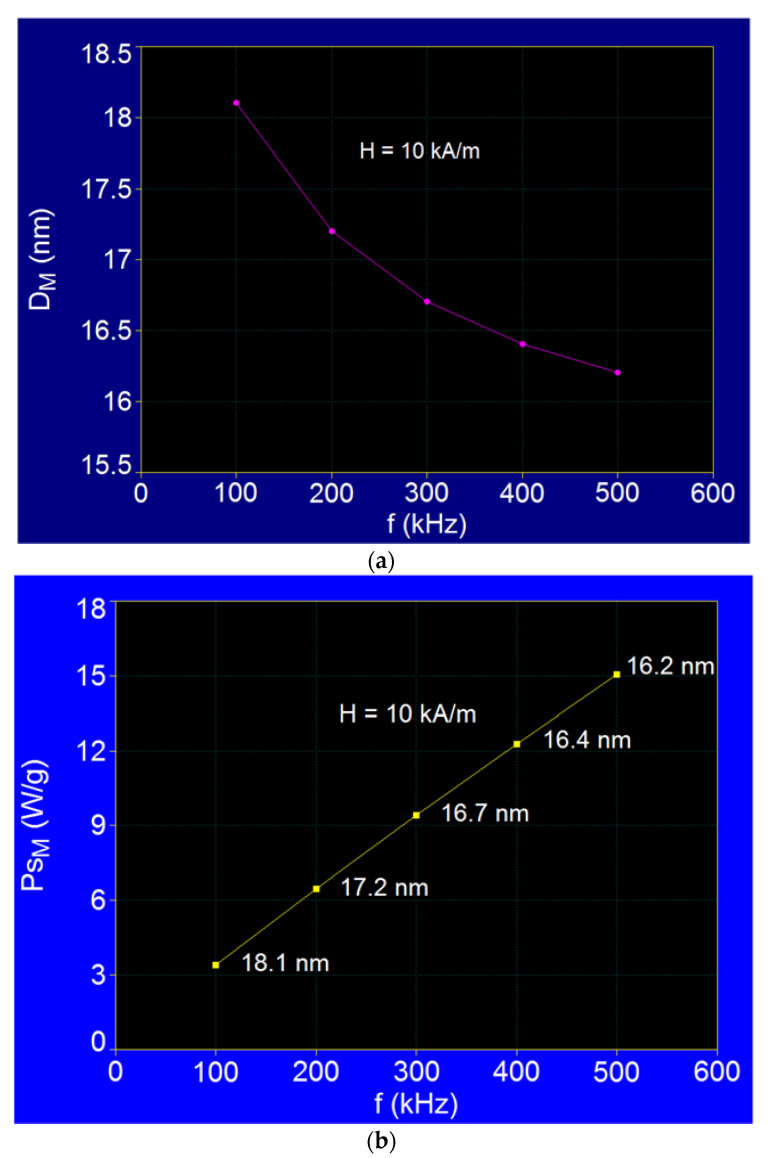
The variations of (**a**) D_M_ and (**b**) P_sM_ as a function of magnetic field frequency for dispersed Fe_3_O_4_-γ-CDs bionanoparticles.

The results obtained depending on the frequency show a shift of the maximum specific loss power towards smaller values of the nanoparticles diameter, respectively from 18.1 nm to 16.2 nm, when the frequency of the magnetic field increases in the range 100–500 kHz (Figure 9a), simultaneously with the almost linear increase of the maximum specific loss power (Figure 9b).

A similar variation in the diameter of nanoparticles D_M_ was observed both in the case of F_3_O_4_ nanoparticles [37] and in the case of CoFe_2_O_4_ nanoparticles [52]. However, in the case of Fe_3_O_4_ nanoparticles coated with γ-CDs it is found that there is a difference between the diameter values corresponding to the maximum specific loss power compared with the case of Fe_3_O_4_ nanoparticles, a difference that increases as the frequency decreases at 100 kHz. If, in the case of Fe_3_O_4_ nanoparticles, the diameters corresponding to the maximums of the specific loss power change in the range of 16.1–17.3 nm [37], in the case of bionanoparticles of Fe_3_O_4_–γ-CDs the diameters corresponding to the maximums change in the range 16.2–18.1 nm when the frequency decreases from 500 kHz to 100 kHz (Table 3, Figure 9a). This difference obtained in the case of bionanoparticles of Fe_3_O_4_–γ-CDs is due to the presence of the organic layer of CDs at the surface of the nanoparticles, which contributes through its thickness to the hydrodynamic diameter and implicitly to the Brown relaxation mechanisms. As a result, the maximum specific loss power shifts to higher values of nanoparticle diameters when the frequency decreases to 100 kHz, when the hydrodynamic diameter increases and thus increases the Brown relaxation time (e.g., at 100 kHz the diameter D_M_ is 18.1 nm for Fe_3_O_4_–γ-CDs bionanoparticles instead of 17.3 nm for F_3_O_4_ nanoparticles [37]).

### 3.4. Maximum Specific Loss Power in Superparamagnetic Hyperthermia with Fe_3_O_4_–γ-CDs Bionanoparticles in Optimal Conditions for the Allowable Limit Frequency

In order to obtain the maximum efficiency in superparamagnetic hyperthermia, it must be optimized. Thus, it is necessary to know under what conditions the maximum specific power can be obtained within the allowable biological limit without affecting the healthy tissues.

**Figure 10:**
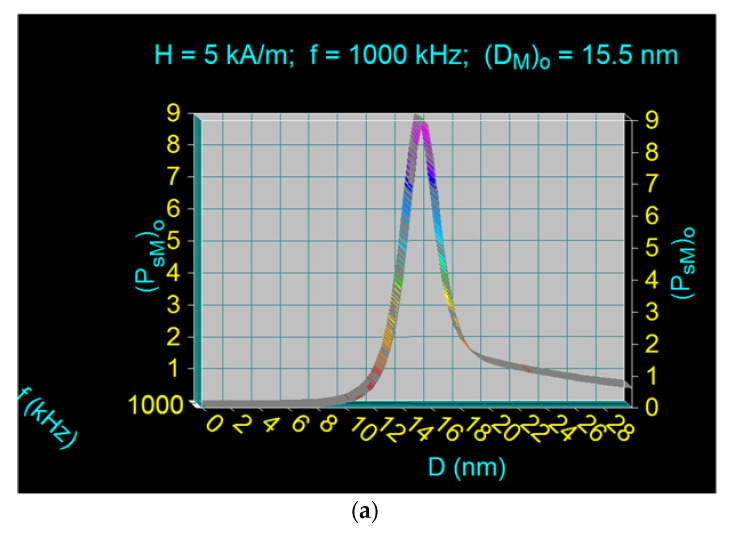

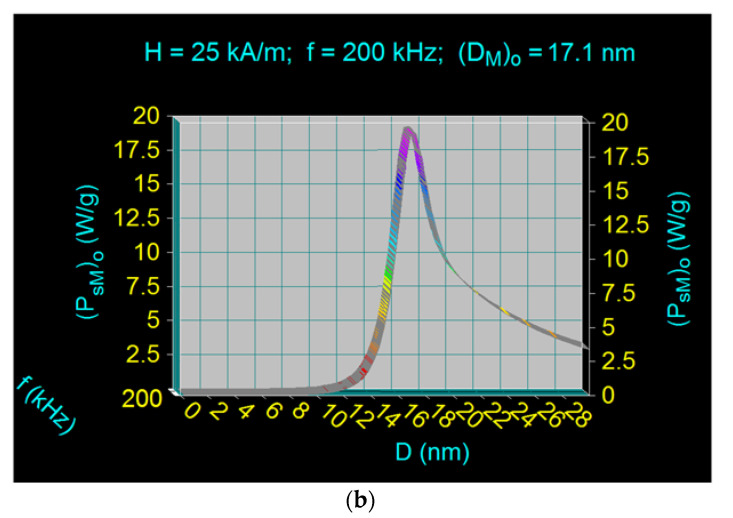
Maximum specific loss power for the allowable biological limit (P_sM_)_o_ at (**a**) 1000 kHz and (**b**) 200 kHz, for Fe_3_O_4_ nanoparticles coated with γ-CDs dispersed in liquid.

Considering the condition established for the parameters of the alternating magnetic field for the biological limit up to which the magnetic hyperthermia can be applied safely [53],

(10) $$H\times f=5\times 10^{9}\;\mathrm{AHz}/m$$

we calculated the maximum specific loss powers in the case of bionanoparticles Fe_3_O_4_–γ-CDs for the allowable limit frequencies in the range 100–1000 kHz. Figure 10 shows the variations of the maximum specific loss powers for the allowable biological limit (P_sM_)_o_ depending on the diameter of the nanoparticle D, for the limit frequencies of 200 kHz and 1000 kHz (in condition (10)). For the limit frequency of 1000 kHz and the magnetic field of 5 kA/m (Figure 10a) the maximum specific loss power is obtained at the optimal diameter of 15.5 nm, being determined by the Néel relaxation of the magnetic moments of Fe_3_O_4_–γ-CDs bionanoparticles. At the limit frequency of 200 kHz and the magnetic field of 25 kA/m (Figure 10b) the maximum specific loss power is obtained at the optimal diameter of 17.1 nm, being determined by both Néel relaxation and Brown relaxation of Fe_3_O_4_–γ-CDs bionanoparticles in approximately equal proportions. At the same time, the maximum specific loss power at the limit frequency of 200 kHz is significantly higher, being 2.14 times higher than the maximum specific loss power obtained at the limit frequency of 1000 kHz. This shows that, practically, it is more advantageous to obtain magnetic hyperthermia at lower frequencies and higher magnetic fields than vice-versa.

The results in Table 4 were obtained by calculating the maximum specific loss powers (P_sM_)_l_ for different limit frequencies (f_l_) and the magnetic fields resulting from the condition given by Equation (10), using diagrams such as those of Figure 10. At the same time, for each maximum of the specific loss power, the corresponding values of the diameters of bionanoparticles were determined, called optimal diameters (D_M_)_o_. The table also specifies the types of relaxations that take place at different frequency limits, mentioning comparatively the proportion of a relaxation process in relation to the other (Néel and/or Brown).

**Figure 11:**
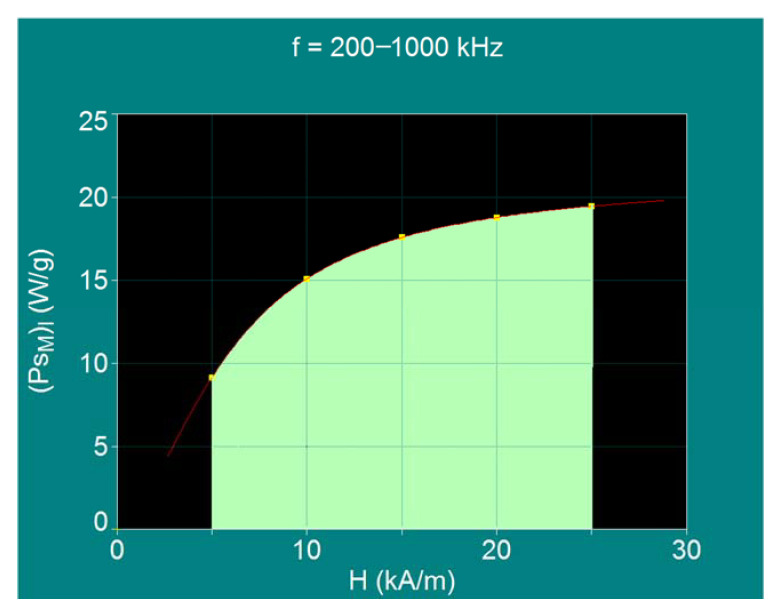
The variation of maximum specific loss power for the allowable biological limit as a function of magnetic field amplitude for Fe_3_O_4_ nanoparticles coated with γ-CDs dispersed in liquid.

Representing graphically the maximum specific loss power for the biological admissible limit (P_sM_)_l_ as a function of the magnetic field H, for the limit frequencies f_l_ (in condition (10)), the red curve in Figure 11 is obtained. According to this result, the optimal recommended range for use in superparamagnetic hyperthermia in the case of the Fe_3_O_4_–γ-CDs biocompatible nanoparticles dispersed in liquid is delimited by light green in the figure.

At higher frequencies than 1000 kHz it is more difficult to achieve superparamagnetic hyperthermia due to the technical difficulties associated with obtaining the magnetic field in the inductor coil (through which intense currents must pass). At the same time, at this frequency the maximum specific loss power decreases significantly. Furthermore, the use of magnetic fields higher than 25 kA/m (at frequencies lower than 200 kHz) is not practically justified because the maximum specific loss power will increase only slightly (Figure 11), going towards a value of saturation for higher magnetic fields.

In conclusion, in order to obtain the maximum efficiency in superparamagnetic hyperthermia with Fe_3_O_4_–γ-CDs bionanoparticles, the optimal conditions established above must be achieved: the magnetic field must be in the range 5–25 kA/m, the frequency in the range 200–500 kHz or even up to 1000 kHz, under the condition given by Equation (10), and the diameter of the bionanoparticles should be in the range 16.2–17.1 nm or even up to 15.5 nm. Thus, the values obtained for the maximum specific loss powers in the range of 5–25 kA/m are sufficient for the rapid heating, necessary in magnetic hyperthermia for the irreversible destruction of tumor cells by apoptosis [54,55], of magnetic nanoparticles [52] to temperatures of 42.5–43 °C.

## 4. Conclusions

In superparamagnetic hyperthermia with Fe_3_O_4_ nanoparticles coated with γ-CDs the specific loss power is determined by both Néel relaxation and Brown relaxation due to the organic layer on the surface of the nanoparticles that determines a hydrodynamic diameter. Néel relaxations are dominant at higher frequencies, around 400–500 kHz, and at lower frequencies, <150 kHz, both Néel and Brown relaxations are present in approximately equal proportions. Due to the presence of the layer of γ-CDs on the surface of Fe_3_O_4_ nanoparticles, the maximum specific loss power obtained in superparamagnetic hyperthermia with bionanoparticles of Fe_3_O_4_–γ-CDs shifts from 18.1 nm to 15.5 nm diameter of the nanoparticles when the frequency changes from 100 kHz to 1000 kHz; the shift of the maximum is not influenced by the amplitude of the magnetic field. However, the values of the nanoparticle diameters at which the maximum loss power are obtained are different from those of the Fe_3_O_4_ nanoparticles, where in the frequency range of 100–1000 kHz the diameters are 17.3 nm at 100 kHz and 15.4 nm at 1000 kHz, the difference being more accentuated at lower frequencies (toward 100 kHz). The different values of the bionanoparticle diameters of Fe_3_O_4_–γ-CDs for which the maximum specific loss power is obtained are due to the organic layer of γ-CDs on the nanoparticle surface, which contributes significantly to the maximum Brown relaxation at lower frequencies (e.g., at 100 kHz the maximum specific loss power is obtained for the diameter of 17.3 nm in the case of Fe_3_O_4_ nanoparticles, and for the diameter of 18.1 nm in the case of bionanoparticles of Fe_3_O_4_–γ-CDs).

The maximum specific loss power obtained in superparamagnetic hyperthermia with nanoparticles of Fe_3_O_4_–γ-CDs increases approximately linearly, both with the increase of the magnetic field in the range of 5–50 kA/m, and with the increase of the frequency in the range of 100–500 kHz.

At low frequencies <250 kHz, due to the presence of Brown relaxation in saline, magnetic hyperthermia may be obtained in a wide range of values for nanoparticle diameters, from 16.9 nm to 25–30 nm. Although the maximum specific loss power decreases to larger diameters, it is still sufficient to heat the nanoparticles to the optimum temperature of 42.5–43 °C required for the destruction of tumor cells by apoptosis. For diameters larger than 18.5–19 nm superparamagnetic hyperthermia is obtained exclusively by Brown relaxation processes, the Néel relaxation processes being negligible in this field of nanoparticle size. This is a major practical advantage in the real case of the existence of nanoparticle size distributions, which are usually obtained in nanoparticle preparation methods, because the nanoparticle diameter in this case is no longer a critical parameter, and must no longer have a strict value (e.g., the value of 17.1 nm at the frequency of 200 kHz, in order to obtain the maximum specific loss power). Thus, at low frequencies, practically wide distributions of nanoparticle sizes can be used to obtain magnetic hyperthermia by Brown relaxation.

Under optimal conditions, for the admissible biological limit for the field and frequency, the maximum specific loss power in superparamagnetic hyperthermia with Fe_3_O_4_–γ-CDs bionanoparticles is obtained for the magnetic field in the range of 10–25 kA/m and a frequency in the range of 200–500 kHz, for diameters of the nanoparticles in the range of 16.2–17.1 nm (Table 4 and Figure 11). At higher frequencies, but in special conditions, the frequency could be increased even up to 1000 kHz and a corresponding field of 5 kA/m, in which case the diameter of the nanoparticles must be 15.5 nm for optimal conditions.

This study allows the practical implementation of superparamagnetic hyperthermia under optimal conditions using biocompatible Fe_3_O_4_ nanoparticles coated with γ-CDs dispersed in liquid in order to obtain maximum efficiency and lack of cellular toxicity.

## Figures and Tables

**Table 1:** The characteristic observables of Fe_3_O_4_–γ-CDs bionanoparticles and the parameters of the alternating magnetic field.

Observables	D (nm)	d (nm)	M_s_ * (kA/m)	K * (kJ/m^3^)	ρ * (×10^3^ kg/m^3^)	ε	η (kg/ms)	f (kHz)	H (kA/m)
Values	1–30	1.6	480	11	5.24	0.024	7 × 10^−4^	100–1000	5–50

* [49,50].

**Table 2:** Maximum specific loss power and diameters corresponding with the maximums in the case of dispersed Fe_3_O_4_–γ-CDs bionanoparticles.

Frequency			500 kHz		100 kHz	
	Observables	H (kA/m)	P_sM_ (W/g)	D_M_ (nm)	P_sM_ (W/g)	D_M_ (nm)
Nr.						
1		10	15.04	16.2	3.38	18.1
2		30	58.32	16.1	12.05	18.0
3		50	101.72	16.1	20.73	18.0

**Table 3:** The values of P_sM_ and D_M_ at different frequencies for dispersed Fe_3_O_4_–γ-CDs bionanoparticles. H = 10 kA/m; 2d = 3.2 nm.

Nanoparticles		Fe_3_O_4_–γ-CDs		
	Observables	f (kHz)	P_sM_ (W/g)	D_M_ (nm)
Nr.				
1		100	3.38	18.1
2		200	6.44	17.2
3		300	9.38	16.7
4		400	12.25	16.4
5		500	15.04	16.2

**Table 4:** The values of maximum specific loss power (P_sM_)_l_ and optimal diameters D_Mo_ for the allowable biological limit, in the case of Fe_3_O_4_ nanoparticles coated with γ-CDs dispersed in liquid.

	Observables	H (kA/m)	f_l_ (kHz)	H × f (AHz/m)	(P_sM_)_l_ (W/g)	(D_M_)_o_ (nm)	Relaxation Type
Nr.							
1		5	1000	5 × 10^9^	9.09	15.5	Neel relaxation prevails
2		10	500	5 × 10^9^	15.04	16.2	Neel >>> Brown
3		15	334	5 × 10^9^	17.57	16.6	Neel >> Brown
4		20	250	5 × 10^9^	18.74	16.9	Neel > Brown
5		25	200	5 × 10^9^	19.44	17.1	Neel and Brown relaxations in proportion approx. equal
6		50	100	5 × 10^9^	20.73	18.1	Brown relaxation prevails

## Data Availability

This is not applicable.
